# 360° Video-Based Virtual Reality for Preparing Medical Students for Body Donor Dissection: Randomized Controlled Trial

**DOI:** 10.2196/95423

**Published:** 2026-06-08

**Authors:** Deema Al-Sheikhly, Khadija Ahmed Elmagarmid, Mohamed Ali Hammami, Soha Roger Dargham, Mange Festo Manyama

**Affiliations:** 1 Continuing Professional Development Division, Medical Education Division, Weill Cornell Medical College in Qatar, Doha, Qatar; 2 Medical Education Division, Weill Cornell Medical College in Qatar, Education City, Al-Luqta Street, P.O. Box 24144, Doha, 24144, Qatar, 974 44928303, 974 44928337

**Keywords:** virtual reality, dissection, medical students, anatomy, educational technology, anxiety, emotions

## Abstract

**Background:**

Body donor dissection is fundamental to medical education but often induces anxiety and emotional distress in students, potentially impacting learning outcomes and well-being. Traditional preparation methods emphasize technical and procedural elements while inadequately addressing students’ emotional challenges. Recent advances in educational technology, particularly 360° video-based virtual reality (VR), may enhance students’ emotional readiness by providing immersive previews of dissection environments. However, the application of this technology specifically for emotional preparation for body donor dissection remains largely unexplored.

**Objective:**

This study aimed to develop and evaluate a 360° video-based VR application designed to enhance medical students’ emotional preparedness for their first body donor dissection experience.

**Methods:**

A randomized controlled longitudinal study was conducted with 43 first-year medical students (26/43, 60.5% female, mean age 20.9, SD 0.57 years) at Weill Cornell Medicine-Qatar in Fall 2025. Participants completed a baseline survey including the 40-item State-Trait Anxiety Inventory and were randomly assigned to intervention (n=22) or control (n=21) groups using computer-generated permuted block randomization. Before their first dissection session, the intervention group viewed a custom-designed 360° video-based VR experience that featured a virtual tour of the anatomy laboratory and a simulated first encounter with a body donor. The control group received no intervention. State-Trait Anxiety Inventory surveys were administered at baseline (survey 1, all participants), post-VR intervention (survey 2, intervention group only), and postfirst dissection (survey 3, all participants). A follow-up perception survey (survey 4) was administered to the intervention group 1 week into the dissection course. Data were analyzed using 2-tailed paired-samples and independent-samples *t* tests, with qualitative responses analyzed using artificial intelligence–assisted thematic analysis.

**Results:**

The intervention group demonstrated a statistically significant reduction in trait anxiety (TA) immediately following the VR experience (mean difference 2.32, SD 4.95; *t*_21_=2.20; *P*=.04), while the reduction in state anxiety (SA) was not significant (mean difference 2.41, SD 8.55; *t*_21_=1.32; *P*=.20). No significant differences in SA or TA were found between intervention and control groups immediately before the first dissection session (SA: *t*_41_=0.03; *P*=.98 and TA: *t*_41_=0.70; *P*=.49) or in anxiety trajectories from baseline to postdissection (SA: *t*_32_=0.85; *P*=.41 and TA: *t*_32_=0.46; *P*=.65). Female students reported higher baseline TA compared to normative college populations (45.42 vs 40.40; mean difference 5.02, SD 7.72; *t*_25_=3.32; *P*=.003). Qualitative analysis revealed positive perceptions, with 91% (10/11) reporting clear content and 82% (9/11) recommending it to future cohorts. Key perceived benefits included environmental familiarization, procedural understanding, and psychological preparation.

**Conclusions:**

The 360° video-based VR intervention significantly reduced TA and was perceived as valuable for emotional and procedural preparation. The intervention shows promise as a preparatory tool for enhancing emotional and procedural readiness; however, its impact on objective educational outcomes was not assessed and warrants further investigation.

## Introduction

Human dissection is a cornerstone of medical education, providing unique insights into human anatomy [1]. It is through the dissection of donated bodies that students are able to have a hands-on approach to understanding the organ systems and their relationship with each other [2]. However, human dissection can be a source of significant stress and anxiety for many students, potentially impacting their learning outcomes and emotional well-being. The emotional and psychological challenges associated with the dissection of donated bodies have been well-documented in the literature, with studies reporting a range of reactions including anxiety, fear, disgust, and even symptoms of posttraumatic stress in some cases [3,4]. Traditional methods of preparation tend to emphasize the technical and procedural elements of dissection, often neglecting to adequately prepare students for the complex emotions that may emerge [5]. Addressing the emotional preparedness of students for dissection is essential to fostering a more supportive and effective learning environment. In response to these challenges, various interventions have been explored to better prepare students emotionally before their first dissection experience. However, findings regarding their effectiveness have been mixed. For example, auditory beat stimulation like binaural beats and the use of background music have been shown to reduce students’ anxiety during dissection sessions [6]. Evidence also suggests that introducing students to the body donor experience through stepwise anatomical demonstrations can reduce mental distress prior to dissection [7]. In contrast, preparatory classes addressing death and dying alongside ethical, religious, social, and family issues associated with dissection did not alleviate stress and, in some cases, increased anxiety among first-year medical students [8].

Recent advances in educational technology and multimedia learning offer promising avenues for enhancing students’ emotional readiness for human dissection. Video-based interventions, in particular, have shown potential in various educational contexts for reducing anxiety and improving learning outcomes [9]. Virtual reality (VR) is also emerging as an invaluable tool in medical education, allowing students to experience clinical environments and interact with the human body in a controlled, nonthreatening, and engaging manner [10,11]. One specific application of VR technology is 360° video-based VR, which immerses learners in realistic, navigable environments that can be explored through head-mounted displays. A 360° video is produced using a specialized camera that records in all directions at once. The captured footage is subsequently stitched together to generate a seamless spherical video, which can be viewed on smartphones, computers, or with a VR headset for enhanced immersion [12]. Recently, 360° video-based VR has been used to prepare students for their clinical clerkships, showing very positive results in enhancing their readiness and confidence [13]. However, the application of this technology specifically to emotional preparation for the dissection of donated bodies remains largely unexplored.

Our research sought to leverage recent technological advancements to assess the real-time impact of VR interventions on students’ anxiety levels. Specifically, this study aimed to develop and evaluate a 360° video-based VR application designed to enhance medical students’ emotional preparedness for their first body donor dissection experience. We hypothesized that students assigned to view the 360° video-based application would exhibit lower anxiety levels before the first dissection compared with those in the control group. By improving emotional preparedness, this approach may help students approach the dissection experience with greater confidence and comfort, which could support their learning experience in anatomy education. The anticipated outcome of this research was a validated intervention that can be implemented broadly to reduce human dissection-related anxiety in medical students.

## Methods

### Participants and Recruitment

The study population comprised first-year medical students who matriculated in Fall 2025 at Weill Cornell Medicine-Qatar (WCM-Q) and were enrolled in the required human anatomy course. Participants were recruited via an invitation email distributed at the start of orientation week, which provided an overview of the study and invited students to participate. An in-person information session was subsequently conducted in a lecture hall on the first day of orientation, during which the study objectives, procedures, and expectations were explained. Throughout the recruitment process, students were explicitly informed that participation was entirely voluntary, independent of anatomy course requirements, and would not affect their academic evaluation or relationships with faculty. Students who expressed interest were provided with consent forms for review and signature.

### Study Design

This study used a randomized controlled longitudinal design. Each consenting first-year medical student was asked to generate a unique code to maintain anonymity before completing a baseline survey (survey 1) administered online via Qualtrics. The baseline survey consisted of the 40-item State-Trait Anxiety Inventory (STAI) and was designed to assess students’ anxiety levels prior to participating in the 360° video-based VR experience and before their first dissection session. Participants were then randomly assigned to either a control or intervention group using computer-generated permuted block randomization (block sizes of 2 and 4).

Later on the same day, students in the intervention group participated in a brief 360° video-based VR experience conducted in small groups in a lecture hall, with each session lasting no more than 10 minutes. The VR experience sessions were facilitated by study investigators to ensure proper guidance and technical support. The control group followed the current standard procedure of no exposure to any intervention.

Two additional STAI-based online surveys were administered at predefined time points: the second (postintervention; survey 2) immediately after the VR session to the intervention group, and the third (postdissection; survey 3) immediately after the first body donor dissection to all participants.

An additional non-STAI–based follow-up survey (survey 4) was administered to students in the intervention group 1 week into the dissection course to evaluate their perceptions of the VR experience and its role in emotional preparedness.

Each survey required no more than 10 minutes to complete.

This study was registered at ClinicalTrials.gov (Identifier: NCT07521033).

**Figure 1.**
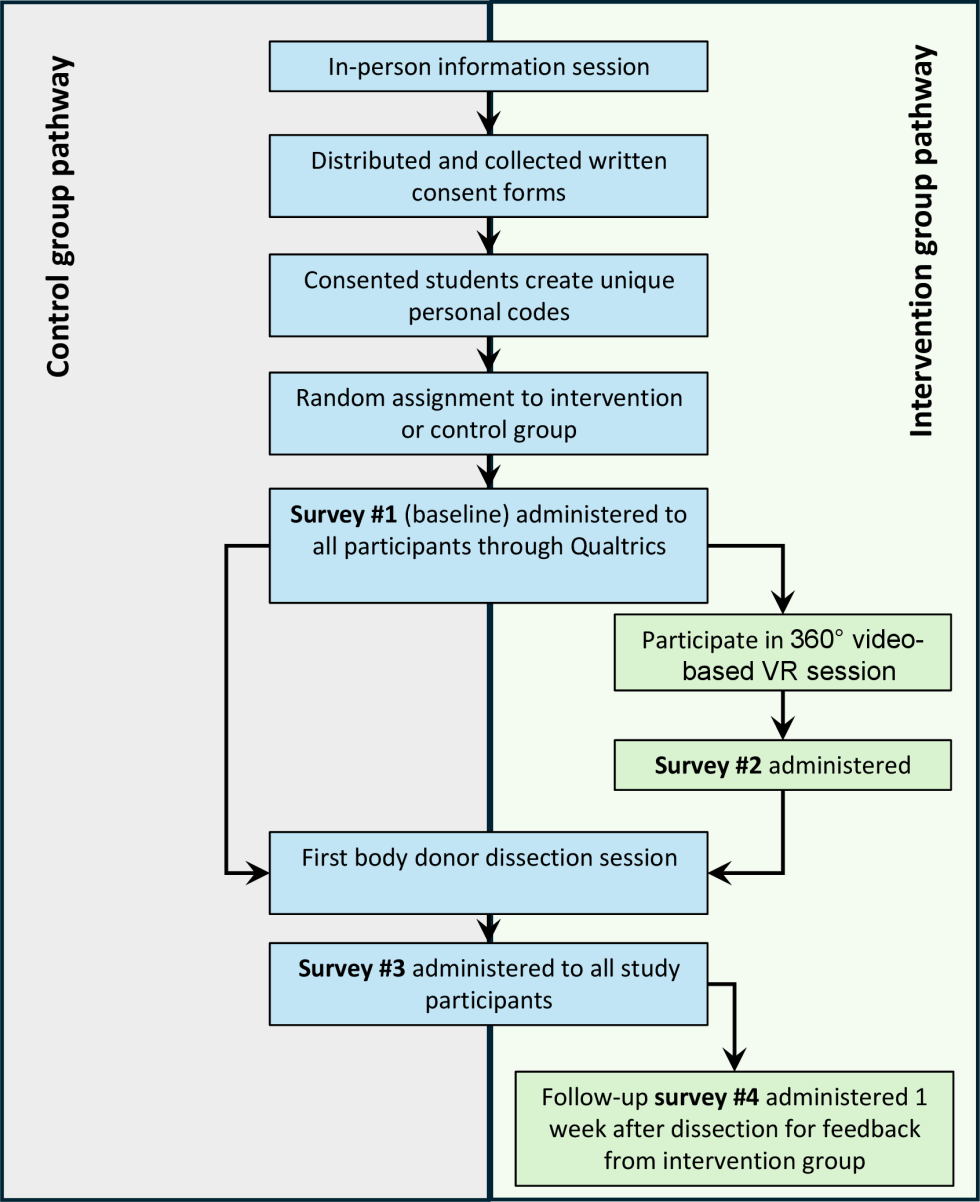
Flowchart of study procedures and assessment time points. Overview of participant allocation and anxiety assessment schedule for control and intervention groups across baseline, predissection, and postdissection time points. VR: virtual reality.

Figure 1 presents a flowchart summarizing the study procedures.

### Study Setting

The gross anatomy course at WCM-Q is delivered during the first year of the medical program and spans one academic semester. Dissection sessions are conducted twice weekly, with each session lasting approximately 2.5 hours. On the first day of the course, students attend a 45-minute introductory lecture on the anatomy of the back and spinal cord, followed by a short break of 10‐15 minutes. After the break, students participate in a session titled *“*Meet your donors*.”* This session begins with a 20-minute lecture outlining the structure of the dissection course, laboratory conduct, and safety rules. Students then proceed to the adjacent anatomy laboratory, where they are introduced to their assigned dissection table, body donor, and group members. Following these introductions, they begin their first dissection session of the back and spinal cord. Dissection groups typically consist of 4 to 5 students and remain unchanged throughout the course.

### Development of the 360° Video-Based VR Experience

The 360° video-based VR experience was developed at WCM-Q by the research team using a commercial 360° camera (Insta360 X-series) that records omnidirectionally through a pair of ultra-wide lenses, simultaneously capturing the full spherical environment. This setup allows for the creation of a video in which viewers can look around the entire scene when viewed on a VR headset. The experience was designed to simulate the sequence of events that students typically encounter during the “Meet Your Donors” session on the first day of cadaver dissection.

To recreate the perspective of a student entering the anatomy laboratory, sensor height was maintained at approximately eye level of the observer. Recordings were also conducted across multiple locations within the anatomy facility, including the laboratory entrance, preparation areas, and the dissection area. The camera remained stationary during most recordings to minimize motion sickness and maintain viewer stability within the VR environment.

Following data capture, the footage was transferred to a workstation for stitching and postprocessing. The dual-lens recordings were automatically stitched into an equirectangular 360° video format, combining the 2 hemispherical images into a spherical video suitable for immersive viewing. The 360° stitched videos were arranged in sequential order to mirror the typical sequence of events encountered during a first anatomy dissection session in the laboratory.

The footage was then imported into 3DVista Virtual Tour Pro, an immersive media authoring platform, where interactive elements were added, including explanatory text, audio narration, instrument hotspots, and navigation controls that allowed users to move through the virtual environment.

The final output was exported as an interactive 360° immersive learning module, compatible with both desktop viewing and VR head-mounted displays. The experience was delivered using Meta Quest 2 standalone VR headsets, which operate without external computers or cables. The wireless headsets allowed students to engage with the virtual content without the burden of a complex setup. Participants viewed the module while seated in a quiet, distraction-free environment, ensuring comfort and stability throughout the immersive session prior to their first body donor dissection.

### Measures

Anxiety levels at different time points were measured using the STAI, an anonymous, self-administered questionnaire designed to assess anxiety in healthy adults [14]. Recognized as the gold standard for adult anxiety assessment, the STAI has been widely implemented in studies involving student populations [15,16]. The instrument contains 40 items measuring two related yet distinct anxiety dimensions: state anxiety (SA) and trait anxiety (TA). SA assesses subjective, transitory feelings of tension, apprehension, and fear experienced in specific situations (such as before a practical dissection session), whereas TA evaluates an individual’s general or baseline emotional state [17]. Each dimension comprises 20 items that generate separate numerical scores. Both SA and TA scores range from 20 to 80, with higher values indicating elevated anxiety levels.

Perceptions of the 360° video-based VR experience and its role in emotional preparedness for human dissection were evaluated using an anonymous online survey administered to the intervention group 1 week into the dissection course. The survey comprised 6 closed-ended Likert scale items and 2 open-ended questions assessing preparedness for dissection, anxiety reduction, clarity, realism, confidence, and overall usefulness. The items were informed by and adapted from questionnaires used in prior studies investigating student perceptions of VR and other technology-enhanced learning tools in anatomy education [6,18,19].

Participants also reported their biological sex (male or female) as part of the demographic data collected. Sex was treated as a binary variable (male or female) based on self-report.

### Statistical Analysis

Data were analyzed using IBM SPSS Statistics (version 30). Descriptive statistics (means and SDs) were calculated for SA and TA scores, stratified by sex and study group (control vs intervention) at each survey time point. Two-tailed paired-samples *t* tests were used to assess within-group changes in SA and TA across survey time points. Two-tailed independent-samples *t* tests were used to examine differences by sex and between the control and intervention groups at baseline and postdissection. Homogeneity of variance was evaluated using the Levene test; when the assumption was met, equal variances were assumed in the *t* test calculations. One-sample *t* tests were conducted to compare baseline anxiety scores, stratified by sex, with normative college population values reported in the STAI manual. A *P* value <.05 was considered statistically significant.

Open-ended survey responses were analyzed using an artificial intelligence (AI)–assisted thematic approach. Anonymous responses were uploaded to Google Gemini AI (Google LLC) to generate preliminary themes and subthemes. The model was prompted to identify recurring patterns in students’ perceptions of the 360° VR experience, including perceived emotional impact, environmental familiarization, procedural understanding, and overall acceptability, and to group responses into coherent themes with representative subthemes. To ensure methodological rigor, the AI-generated outputs were not used in isolation. All available open-ended responses from participants who completed the qualitative survey (n=11/22) were included in the analysis. All preliminary codes and themes were independently reviewed by 4 members of the research team and compared directly with the original participant responses. Codes were refined, merged, or reclassified as needed through an iterative process. Discrepancies in interpretation were discussed among the authors and resolved through consensus. Final themes were agreed upon only after ensuring that they accurately reflected the underlying meaning and variability of the data.

To address the potential risk of algorithmic bias inherent in large language models, the study acknowledged that Gemini is developed under a responsible AI framework that includes rigorous dataset curation, fairness evaluations, and safety filters specifically designed to mitigate harmful or demographic bias. In addition, reliance was placed on human validation and critical appraisal of AI-generated outputs to minimize the risk of misinterpretation or overgeneralization. These internal safeguards, combined with the subsequent human-led validation and investigator consensus, were used to ensure the integrity and neutrality of the thematic findings.

### Ethical Considerations

This study was reviewed and approved under expedited review by the Institutional Review Board of Weill Cornell Medicine-Qatar (IRB protocol number 25‐00015). All study procedures were conducted in accordance with the ethical standards of the institutional research committee and the principles outlined in the Declaration of Helsinki.

Participation in the study was entirely voluntary. All participants were provided with detailed information about the study objectives, procedures, and expectations during recruitment and an in-person information session. Written informed consent was obtained from all participants prior to enrollment.

To ensure privacy and confidentiality, no personally identifiable information was collected. Participants generated unique codes to anonymize their responses across survey time points. All data were stored securely and accessed only by members of the research team for analysis purposes.

Upon completion of data collection, each participant received a gift voucher valued at approximately US $50 as compensation for their time and participation.

## Results

### Overview

**Figure 2.**
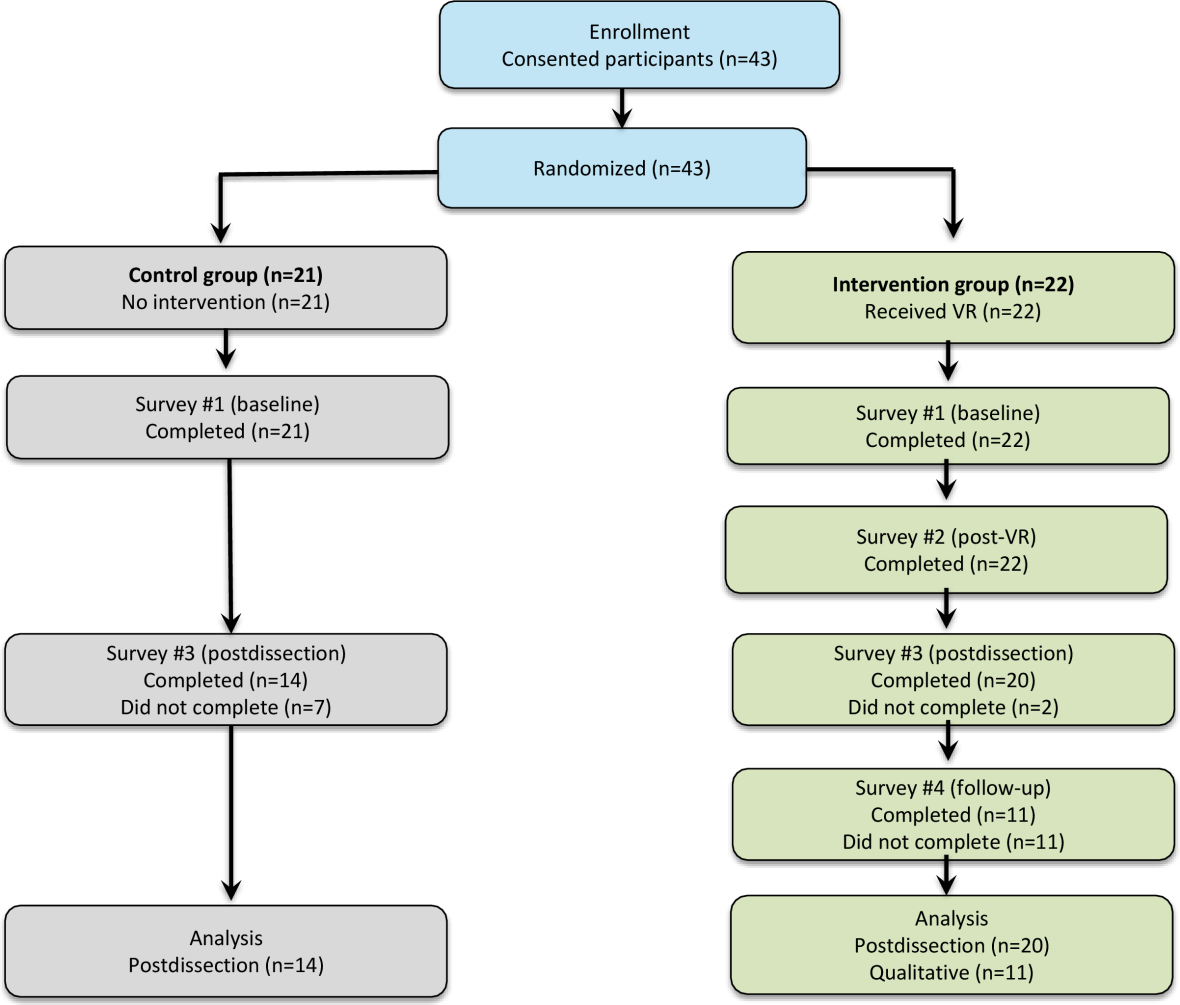
Flow diagram of participant progression through the study. The diagram illustrates enrollment, randomization, allocation to intervention and control groups, follow-up across survey time points, and inclusion in quantitative and qualitative analyses, including attrition at each stage. VR: virtual reality.

A total of 43 students participated in this study, representing approximately 80% (43/54) of the class cohort. Figure 2 shows the flow of participants through the study, including enrollment, randomization, allocation to intervention and control groups, follow-up at each survey time point, and inclusion in the final analysis.

### Demographics

The mean age was 20.9 (SD 0.57) years. Of the 43 participants, 26 (60.5%) were female and 17 (39.5%) were male. Nearly two-thirds (29/43, 67.5%) had no prior exposure to human body donors (Table 1).

**Table 1.** Demographic characteristics of study participants.

Characteristics	Participants
Sex, n (%)	
Male	17 (39.5)
Female	26 (60.5)
Previous body donor exposure, n (%)	
Yes	14 (32.5)
No	29 (67.5)

### Baseline Mean Anxiety Levels

The mean SA and TA levels were higher in females than in males prior to the 360° video intervention and first dissection session. However, 2-tailed independent-samples *t* tests indicated that these differences were not statistically significant. Mean SA levels were 40.73 (SD 10.05) in females and 37.53 (SD 12.46) in males (*t*_41_=−0.90; *P*=.36). Similarly, mean TA levels were 45.42 (SD 7.72) in females and 41.76 (SD 9.44) in males (*t*_41_=−1.33; *P*=.17; Table 2).

**Table 2.** Baseline mean anxiety levels.

Variables and sex	Mean (SD)	*t* test (*df*)	*P* value
SA^a^		−0.90 (41)	.36
Male (n=17)	37.53 (12.46)		
Female (n=26)	40.73 (10.05)		
TA^b^		−1.33 (41)	.17
Male (n=17)	41.76 (9.44)		
Female (n=26)	45.42 (7.72)		

a SA: state anxiety.

b TA: trait anxiety.

### Comparison of Anxiety Levels Before and After the 360° Video-Based VR Intervention

A 2-tailed paired-samples *t* test was conducted to determine whether SA and TA levels differed significantly within the intervention group between baseline (survey 1) and the post-360° VR intervention assessment (survey 2). Both SA and TA scores decreased following the intervention (Table 3). The mean SA score declined from 40.59 (SD 12.23) at baseline to 38.18 (SD 11.68) post intervention; however, this reduction was not statistically significant.

**Table 3.** Comparison between baseline and postintervention anxiety levels among the intervention group.

Variables	Baseline, mean (SD)	Postintervention, mean (SD)	Mean change (SD)	*t* test (*df*)	*P* value
SA^a^ (n=22)	40.59 (12.23)	38.18 (11.68)	2.41 (8.55)	1.32 (21)	.20
TA^b^ (n=22)	44.18 (9.32)	41.86 (9.76)	2.32 (4.95)	2.20 (21)	.04

a SA: state anxiety.

b TA: trait anxiety.

(mean difference 2.41, SD 8.55; *t*_21_=1.32; *P*=.20). In contrast, TA scores showed a statistically significant decrease (mean difference 2.32, SD 4.95; *t*_21_=2.20; *P*=.04).

### Comparison of Anxiety Levels Between Groups Before the First Dissection Session

To assess the impact of the 360° video-based VR experience on anxiety levels prior to the first dissection session, anxiety levels in the control group at baseline (survey 1) were compared with those in the intervention group after 360° video-based VR exposure (survey 2), with both measurements obtained before the first dissection session. Two-tailed independent-samples *t* tests revealed no statistically significant differences between groups. Mean SA levels were 38.29 (SD 9.79) in the control group and 38.18 (SD 11.68) in the intervention group (*t*_41_=0.03; *P*=.98; Table 4). Similarly, mean TA levels did not differ significantly between groups (control: 43.76, SD 7.84; intervention: 41.86, SD 9.76; *t*_41_=0.70; *P*=.49).

**Table 4.** Mean anxiety levels in control (baseline) and intervention (post-360° video) groups prior to first dissection session.

Variables and groups	Mean (SD)	*t* test (*df*)	*P* value
SA^a^		0.03 (41)	.98
Control (n=21)	38.29 (9.79)		
Intervention (n=22)	38.18 (11.68)		
TA^b^		0.70 (41)	.49
Control (n=21)	43.76 (7.84)		
Intervention (n=22)	41.86 (9.76)		

a SA: state anxiety.

b TA: trait anxiety.

### Between-Group Comparison of Change in Anxiety Levels From Baseline to Postdissection

Changes in anxiety levels from baseline (survey 1) to after the first dissection session (survey 3) were compared between the control and intervention groups using 2-tailed independent-samples *t* tests (Table 5). The reduced sample size for this analysis (control: n=14; intervention: n=20) reflects participants who did not complete the postdissection survey (survey 3), and analyses were conducted on complete cases only. Mean change scores represent the difference in anxiety levels across this period, with positive values indicating an increase and negative values indicating a decrease in anxiety. The intervention group demonstrated slight reductions in both SA (mean −0.60, SD 15.14) and TA (mean −1.37, SD 6.63), whereas the control group showed a slight increase in SA (mean 3.36, SD 10.49) and a minimal decrease in TA (mean −0.36, SD 5.88). However, independent-samples *t* tests indicated that the differences in mean change between groups were not statistically significant for either SA (*t*_32_=0.85; *P*=.41) or TA (*t*_32_=0.46; *P*=.65). These findings indicate that, despite within-group improvements, the intervention did not demonstrate superiority over the control group in reducing anxiety prior to the first dissection session. However, given the reduced sample size and variability in responses, these nonsignificant findings should be interpreted cautiously, as the study may have been underpowered to detect small-to-moderate between-group differences.

**Table 5.** Comparison of the change in anxiety levels between control and intervention groups between baseline and postdissection.

Variables and groups	Mean change (SD)	*t* test (*df*)	*P* value
SA^a^		0.85 (32)	.41
Control (n=14)	3.36 (10.49)		
Intervention (n=20)	−0.60 (15.14)		
TA^b^		0.46 (32)	.65
Control (n=14)	−0.36 (5.88)		
Intervention (n=20)	−1.37 (6.63)		

a SA: state anxiety.

b TA: trait anxiety.

### Comparison of Cohort Baseline Anxiety Levels to Normative College Population Data

Baseline anxiety levels of our medical student cohort (survey 1) were compared with established normative values for college students reported in the STAI manual using 2-tailed one-sample *t* tests (Table 6). Female medical students demonstrated significantly higher baseline TA levels (mean 45.42, SD 7.72) compared with the female normative mean of 40.40 (mean difference 5.02; *t*_25_=3.32; *P*=.003). No statistically significant differences from normative values were observed for male participants or for SA in either sex (all *P*>.05).

**Table 6.** Baseline anxiety levels compared to normative college values by sex.

Variables and sex	Normative mean (SD)	Baseline mean (SD)	Mean difference	*t* test (*df*)	*P* value
SA^a^					
Male (n=17)	36.47	37.53 (12.46)	1.06	0.35 (16)	.73
Female (n=26)	38.76	40.73 (10.05)	1.97	1 (25)	.33
TA^b^					
Male (n=17)	38.30	41.76 (9.44)	3.47	1.51 (16)	.15
Female (n=26)	40.40	45.42 (7.72)	5.02	3.32 (25)	.003

a SA: state anxiety.

b TA: trait anxiety.

### Perceived Effectiveness of the 360° Video-Based VR Experience for Emotional Preparedness: Quantitative Findings

**Figure 3.**
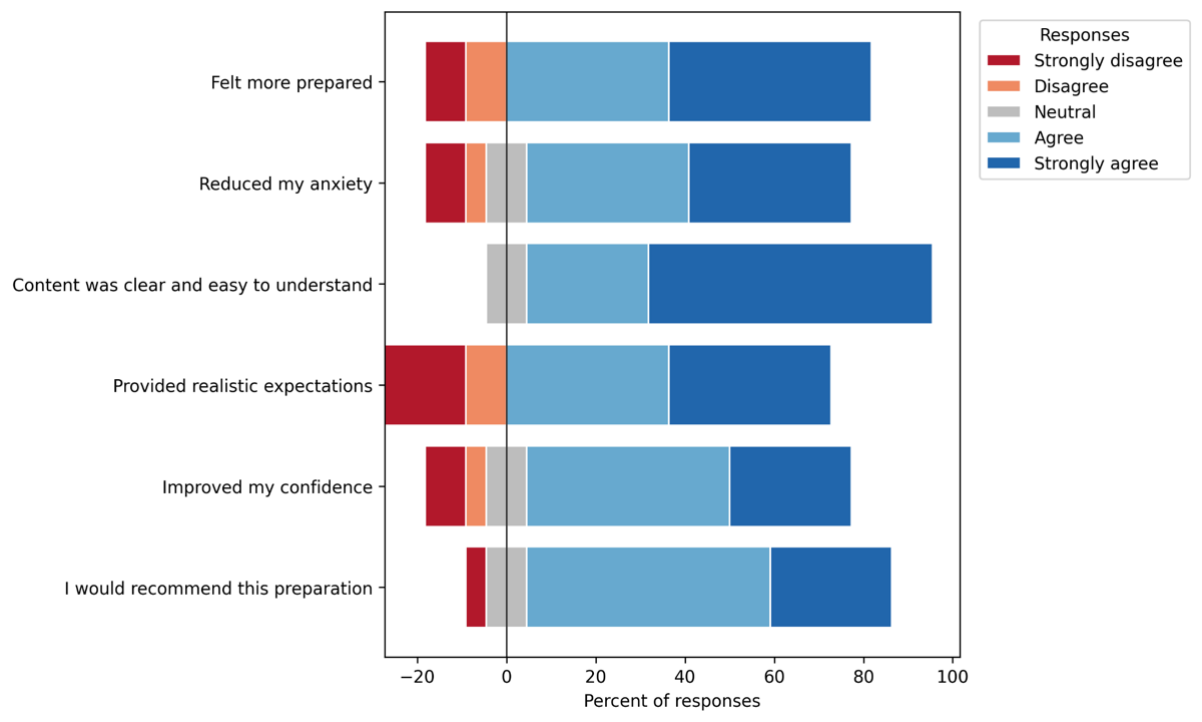
Diverging stacked bar chart of participants’ perceptions of the 360° video-based virtual reality preparation. Follow-up survey results were completed one week into the dissection course, evaluating the perceived usefulness of the 360° virtual reality experience in preparing for the first body donor dissection.

Survey 4 assessed participants’ perceptions of the 360° video-based VR experience and its role in emotional preparedness for the first dissection encounter (Figure 3). Overall, 11 (50%) participants completed the survey. The intervention was overwhelmingly perceived as a positive and effective preparatory tool. The majority of respondents agreed or strongly agreed with all 6 positive statements. The most pronounced agreement was with the clarity of the video content, with 10 out of 11 (91%) students agreeing or strongly agreeing that the content was easy to understand. Most students reported that the intervention helped them feel more prepared and confident in approaching body donor dissection. About 82% (9/11) of students indicated they would recommend this VR preparation to future cohorts.

### Qualitative Exploration: Thematic Analysis

#### Overview

Thematic analysis of the open-ended responses identified 2 main themes with associated subthemes, as shown in Table 7.

**Table 7.** Themes and subthemes from open-ended questions.

Themes	Subthemes
Perceived benefits of the 360° VR^a^ experience	Environmental familiarization Procedural understanding Psychological preparation
Recommendations for enhancement	Inclusion of dissection content Technical improvements

a VR: virtual reality.

#### Theme One: Perceived Benefits of the 360° Video-Based VR Experience

This theme describes participants’ experiences with the 360° video-based VR intervention and their perceptions of its effectiveness. Three subthemes emerged: environmental familiarization, procedural understanding, and psychological preparation.

##### Environmental Familiarization

Most participants valued previewing the anatomy laboratory layout before their first dissection. This advanced exposure reduced uncertainty about entering an unfamiliar space by providing visual familiarity that appeared to serve as an important psychological buffer against the stress of encountering the dissection environment for the first time.

It was nice to know what the lab looks like and the setting of the cadaver.

[P01]

Knowing what the room looked like.

[P03]

To expect how the room looked like. This helped me calm down a bit.

[P10]

##### Procedural Understanding

Participants valued the step-by-step orientation provided by the VR experience, which helped them understand logistical and procedural aspects of the laboratory session, thereby increasing confidence and reducing anxiety about being unprepared.

The order of getting into the anatomy lab was especially helpful knowing where to go for any presentations, our lockers and changing room and signing in.

[P05]

It helped me navigate through what to expect and what to do inside the anatomy lab.

[P09]

##### Psychological Preparation and Visualization

Participants reported that the VR experience helped them mentally and psychologically prepare for the emotional and visual reality of working with human body donors, filling an important gap in the absence of other preparatory experiences. However, one participant found the intervention unhelpful.

It was helpful because we had no other introduction to the anatomy lab and like how it would be so this helped like mentally prepare me for it.

[P07]

I liked how we could see the cadaver clearly in the VR session as that helped me visualize what it would be like in real life.

[P08]

Wasn't that helpful.

[P06]

### Theme Two: Recommendations for Enhancement

This theme describes suggestions for improving the VR intervention. Two subthemes emerged: inclusion of dissection content and technical improvements.

#### Inclusion of Dissection Content

Multiple participants desired actual dissection demonstrations within the VR experience, recognizing that procedural exposure would provide more comprehensive preparation than environmental orientation alone. One participant suggested making such content optional to accommodate varying comfort levels.

The video didnt have any dissection so it didnt really prepare us for what we were going to see.

[P02]

Maybe do an actual dissection if participants are comfortable enough to go ahead with it.

[P03]

Maybe have a section showing actual dissection as it would prepare students better.

[P04]

#### Technical and Delivery Improvements

Participants suggested enhancements to the technical aspects and presentation format of the intervention, indicating that while the VR experience was valuable, improvements in technical delivery and content structure would be beneficial.

To be honest I don't think the VR portion of it is necessary. I think a video would have had the same impact.

[P05]

Maybe subtitles could help.

[P06]

## Discussion

### Principal Findings

This study developed and evaluated a 360° video-based VR application designed to enhance medical students’ emotional preparedness for their first dissection session. The application aimed to familiarize students with the anatomy laboratory environment, provide an understanding of what to expect procedurally, and support their psychological readiness ahead of this experience. The application was not intended to teach dissection techniques or gross anatomy content, but rather to serve as a preparatory tool for students approaching this significant milestone in their medical training.

Our findings suggest a varied impact of the intervention, offering meaningful insights for improving anatomy teaching practices and enhancing student well-being. Comparison of levels of anxiety before and after the 360° video-based VR intervention revealed a statistically significant reduction in trait anxiety immediately post intervention. In contrast, state anxiety showed a nonsignificant reduction. Importantly, while these within-group findings suggest a beneficial effect, the absence of statistically significant between-group differences indicates that the intervention did not demonstrate clear superiority over the control condition. This pattern suggests that the VR intervention may contribute to reductions in anticipatory or baseline anxiety (trait anxiety), while its effect on acute situational anxiety (state anxiety) remains less clear. However, given the absence of statistically significant between-group differences, these findings should be interpreted cautiously, as the study may not have been sufficiently powered to detect subtle intervention effects. While the decrease in SA did not reach statistical significance, the downward trend suggests potential anxiety-reducing effects that may become more pronounced with further refinements to the intervention. Although students in the intervention group experienced a significant reduction in TA following the VR experience, between-group comparisons immediately before the first dissection session showed no significant differences in either SA or TA levels when compared to the control group. These findings highlight that the current form of the intervention is effective for familiarization and uncertainty reduction, rather than directly mitigating visceral, in-the-moment anxiety. Similarly, the trajectory of anxiety for each group from baseline to postdissection showed no significant between-group differences; however, the intervention group demonstrated slight reductions in both SA and TA, while the control group showed a slight increase in SA.

The significant reduction in TA levels in the intervention group immediately post intervention is particularly noteworthy, as it suggests that the 360° video-based VR intervention may positively influence students’ baseline emotional tendencies in high-stress learning contexts, even if immediate situational anxiety remains more resistant to change. However, trait anxiety is generally considered a relatively stable psychological construct, and changes observed over a short time frame should be interpreted with caution. In this context, the observed reduction may reflect a temporary shift in perceived baseline anxiety or context-specific emotional readiness rather than a true modification of stable personality traits. This interpretation is further supported by the qualitative feedback, where students indicated that while the VR experience helped them understand procedural and environmental expectations, it did not replicate the actual dissection content. These findings are further supported by existing literature, which suggests that structured preexposure interventions designed to foster a sense of predictability and control in unfamiliar environments are effective in reducing trait anxiety [4,15,20].

A substantial body of research supports the use of interventions to alleviate anxiety associated with cadaveric dissection. Böckers et al [7] found that a step-by-step introduction of dissection through anatomical demonstrations of organ systems helped to reduce the mental distress of students. The observed reduction in mental distress may have occurred because the intervention helped students acclimatize early, familiarizing them with body donors and the dissection process before they entered the anatomy laboratory. ABS, such as binaural beats and background music, has also shown significant efficacy in reducing state anxiety during dissection sessions [6,21]. The anxiety-reducing effects of binaural beats likely operate through multiple mechanisms, including anxiolytic properties [22], cognitive enhancement [23], and improved psychophysiological states [24]. In particular, theta-frequency binaural beats have been shown to elicit relaxation responses [25].

While our 360° VR intervention builds upon the approaches described in the aforementioned studies on anxiety reduction in body donor dissection, it offers distinct advantages. These include standardization and immersive environmental familiarization without requiring prior laboratory access. By providing environmental familiarization and procedural awareness, the VR intervention bridges the gap between traditional technical preparation and the complex emotional reality of the anatomy laboratory. Unlike preparatory classes on death and dying, which have occasionally increased stress levels [8], the VR approach offers controlled exposure that fosters psychological preparation.

The use of an immersive 360° VR intervention in our study is supported by recent research demonstrating its value in preparing learners for clinical practice. Pieterse et al [13] reported that 360° VR applications enhanced students’ readiness and confidence for clinical clerkships by allowing them to explore realistic clinical scenarios in a safe, nonthreatening environment prior to in-person exposure. Similarly, a recent study found that an immersive 360° VR-based module improved students’ understanding and confidence in clinical decision-making for obstetric emergencies by providing a user-friendly learning environment [26]. Electroencephalography studies indicate that anxiety is characterized by increased power in higher frequency bands (such as beta waves) and decreased power in lower frequency bands (such as alpha and theta waves) [27,28]. Anxiety-reducing interventions, such as VR and progressive muscle relaxation, have been shown to be associated with decreased beta activity and increased alpha and theta activity [29,30].

The lack of statistically significant differences in SA and TA between the intervention and control groups before the first dissection session may reflect several factors, including both the limited scope of the intervention and the possibility of insufficient statistical power to detect modest effects. First, the 360° VR intervention was brief and focused primarily on environmental and procedural orientation rather than on direct exposure to dissection content. As a result, the intervention was less likely to influence acute state anxiety. While participants valued this orientation, its limited scope may not have sufficiently addressed the visceral and emotional reactions elicited by actual body donor exposure. Second, anxiety before the first dissection is a complex response influenced by prior experiences, personal coping mechanisms, and other factors beyond environmental familiarity. This aligns with findings from previous studies, which have reported that even students with prior dissection experience continue to experience anxiety [31,32], suggesting that procedural knowledge alone does not fully alleviate emotional responses to body donors.

The sex differences in anxiety observed before and after the 360° VR intervention, with female students reporting higher state and trait anxiety than males, are consistent with prior literature demonstrating that female students typically experience greater anxiety and stress across both general and medical education populations [4,7,20,33,34]. In our cohort, female students also exhibited significantly higher trait anxiety scores than normative college-aged female populations. Because trait anxiety reflects a stable predisposition to perceive situations as threatening, elevated levels may increase vulnerability to stress in emotionally demanding learning environments such as human dissection. This heightened baseline anxiety may additionally be influenced by contextual and sociocultural factors specific to the Middle Eastern region. Studies from Qatar and the broader Middle East report relatively high levels of anxiety among university students, with females consistently demonstrating greater psychological distress than males [35-37]. Several contributing factors have been reported, including academic pressures, sex-related societal expectations, and stigma related to mental health, which have been suggested to limit coping and help-seeking behaviors [35,36]. Together, these influences may predispose female students to elevated dispositional anxiety even before encountering course-specific stressors. Although our study did not identify differential intervention effects by sex, future research should explore whether particular subgroups may derive greater benefit from preparatory strategies such as VR-based interventions.

The qualitative findings highlight a crucial preparatory dimension beyond anxiety reduction, with students reporting overwhelmingly positive perceptions of the VR experience. However, these results are based on only 50% (11/22) of participants, introducing potential nonresponse bias; students who responded may have had stronger positive impressions than nonresponders, and interpretations of the qualitative feedback should be made cautiously. Students indicated that the 360° VR experience helped them understand what to expect regarding the physical environment, procedural steps, and logistical aspects of the dissection session. This aligns with the Uncertainty Reduction Theory, which postulates that much of the anxiety associated with novel experiences stems from uncertainty about what will occur [38]. In this context, psychological preparation appears to arise from reducing cognitive uncertainty by familiarizing oneself with the anatomy laboratory environment and procedural processes. This may explain why participants reported feeling more prepared and oriented despite the absence of a statistically significant reduction in state anxiety, suggesting that cognitive readiness and emotional anxiety may be influenced through partially distinct mechanisms. By providing students with a clear mental model of the dissection laboratory and session structure, the 360° VR experience may have addressed cognitive uncertainty even when emotional anxiety remained elevated. Key benefits that emerged included environmental familiarization, procedural understanding, and psychological preparation, mirroring findings from VR-based preparatory studies in clinical training and educational settings [13,39]. The majority of participants found the VR content clear and understandable, and most indicated that they would recommend it to future cohorts. The disconnect between statistical significance and perceived value suggests that the intervention’s benefits extend beyond anxiety reduction alone.

In addition, the qualitative feedback provided valuable insights for refining the 360° VR experience. Students most frequently recommended incorporating actual dissection content rather than limiting the experience to environmental orientation and procedural information. While such content may better prepare students by reducing the shock of first exposure to anatomical structures, it could also overwhelm those who are not yet emotionally ready, potentially increasing anxiety. A practical solution may be a tiered design, allowing learners to select their preferred level of exposure, for example, a basic orientation module and an optional advanced module that includes dissection demonstrations. Such a tiered or opt-in design could be structured progressively, beginning with low-intensity exposure (eg, laboratory environment and procedural overview), followed by intermediate modules (eg, introduction to instruments and handling of body donors), and culminating in optional high-intensity modules featuring actual dissection content. This approach would allow students to self-regulate their exposure based on their emotional readiness and learning needs, thereby supporting both psychological comfort and educational preparedness. Importantly, this personalized progression may enhance the intervention’s ability to address both cognitive uncertainty and, potentially, state anxiety in future iterations.

Despite promising qualitative feedback, this study has several limitations. The modest sample size from a single institution limits generalizability. Additionally, given that only 11 of 22 participants completed the qualitative survey, the potential for nonresponse bias is acknowledged, and the qualitative findings should be interpreted with caution, as respondents may have been more likely to report positive perceptions. Furthermore, the timing of our postintervention and postdissection assessment (immediately after VR intervention and immediately after the first dissection session) may have been suboptimal, as students experience varying anxiety trajectories, with some experiencing initial spikes followed by rapid habituation and others experiencing delayed responses. Longer-term follow-up would better capture the intervention’s sustained effects. A further methodological limitation relates to the timing of measurements between groups. The control group did not undergo an assessment equivalent to the postintervention (survey 2) measurement used in the intervention group. As a result, comparisons between the control group’s baseline and the intervention group’s post-VR scores may be influenced by expectation or anticipation effects, rather than reflecting the isolated impact of the intervention. Although this design allowed for assessment of the immediate effect of the VR experience, it limits the ability to fully attribute observed differences to the intervention itself. Future studies should incorporate parallel measurement time points in both groups to better control for expectation-related influences and strengthen causal inference.

The interpretation of changes in trait anxiety should also be approached with caution. Although a significant reduction was observed within the intervention group, trait anxiety is generally considered a relatively stable construct. The observed changes may therefore reflect short-term, context-specific shifts rather than sustained changes in underlying disposition. Finally, the study focused exclusively on anxiety and perceived preparedness without assessing actual learning outcomes or dissection performance. Whether reduced anxiety translates into improved anatomical learning or engagement remains unclear.

### Conclusions

In conclusion, this study provides evidence that a 360° video-based VR experience can serve as a valuable preparatory tool for medical students facing their first body donor dissection, particularly through enhancing environmental and procedural familiarity. The intervention showed promise through positive student perceptions and a significant within-group reduction in trait anxiety; however, it did not demonstrate statistically significant superiority over the control group in reducing anxiety prior to dissection. While its impact on state anxiety was limited in this iteration, the reduction in trait anxiety and positive student feedback indicate that the intervention effectively supports familiarization and uncertainty reduction. Compared with traditional audiovisual aids and other anxiety-focused strategies, immersive VR provides visual and spatial orientation to the laboratory, reducing uncertainty by allowing learners to virtually explore the space before in-person exposure. With further refinement and research, particularly through future iterations incorporating actual dissection content that may enhance its impact on in-the-moment anxiety, VR-based preparation could become a supportive component of a holistic approach to student well-being in the anatomy laboratory; however, further research is required to determine its impact on educational outcomes.

## Supplementary material

10.2196/95423 Checklist 1 CONSORT-eHEALTH checklist.

## Abbreviations

AI: artificial intelligence
SA: state anxiety
STAI: State-Trait Anxiety Inventory
TA: trait anxiety
VR: virtual reality
WCM-Q: Weill Cornell Medicine – Qatar
